# Differential diagnosis of chronic monoarthritis in chidren

**DOI:** 10.1186/1546-0096-12-S1-P207

**Published:** 2014-09-17

**Authors:** Cristina Battagliotti, Ileana Villarruel

**Affiliations:** 1 Santa Fe, Hospital de Niños Dr Orlando Alassia, Santa Fe, Argentina

## Introduction

Juvenil chronic arthrits (JCA) is the most common chronic rheumatic disease in children an important cause of disability. When only one joint is involved it may be difficult to make an early diagnosis. A detailed history and clinical examination is important to reach a correct diagnosis and appropiated treatment.

## Objectives

To investigate the diagnosis of chronic monoarthritis.

## Methods

Data were collected retropectively for 69 consecutive chronic monoarthritis seen in our hospital during 2000 -20013. Minimal duration of arthritis :3 months 10 patients were excluded for not having complete information.

## Results

There was two age more frequently 2 and 11 years (R: 0.10-14.4) .41 fem,27masc.

The most commont joints involved were:52 knee (88%),5 ankle, 1 elbow and 1 finger.

24(40.6%) patients had JCA, 13 fem 11 masc, the evolution was: 10 pauciarticular, 5 polyarticular 3 Spondyloarthropathies, 6 others. The men age of JAC was 7 years (r:6m-14a). Ocular examination: 1 blefaritis, 1 corneal erosions,1 acute iridiociclitis.

Joint fluid: 90% inflammatory.

Arthroscopic with patology anatomic: 11 nonspecific chronic synovitis.

Another diagnosis were: 5 body forein (pine needle, sliver, toothpick), 6 discoid meniscus, 3 synovial chondromatosis, 2 distention ligament, 2 osteochondritis, 2 synovial cyst, 1 Synovial haemangioma, 1 Pigmented villo-nodular synovitis, 1 patient with agamaglobulinemia (Brutton Disease), 1 Tenosinovitis in Celiac disease.

The laboratory tests include antinuclear antibody and ocular examination were non significative to diferenciate ACJ to another crhronic monoarthritis (Fisher exact test, p> 0.27). On the other hand highly significant association between patients with ACJ and cronic synovitis (X^2^ p>0.3005) was found.

## Conclusion

40.6% of patients with chronic monoarthritis had ACJ, being the most frequent form: pauciarticular. MRI and the synovial biopsy atroscopia play an important role in the diagnosis of a child that presents a chronic monoarthritis.

## Disclosure of interest

None declared.

